# Recovery of Degraded-Beyond-Recognition 19^th^ Century Daguerreotypes with Rapid High Dynamic Range Elemental X-ray Fluorescence Imaging of Mercury L Emission

**DOI:** 10.1038/s41598-018-27714-5

**Published:** 2018-06-22

**Authors:** Madalena S. Kozachuk, Tsun-Kong Sham, Ronald R. Martin, Andrew J. Nelson, Ian Coulthard, John P. McElhone

**Affiliations:** 1 The University of Western Ontario, The Department of Chemistry, 1151 Richmond Street, London, Ontario, N6A 5B7 Canada; 2 The University of Western Ontario, The Department of Anthropology, 1151 Richmond Street, London, Ontario, N6A 5C2 Canada; 3 Canadian Light Source Inc., 44 Innovation Boulevard, Saskatoon, SK S7N 2V3 Canada; 4 National Gallery of Canada, Musée des beaux-arts du Canada, 380 Sussex Drive, P.O. Box 427, Station A, Ottawa, Ontario, K1N 9N4 Canada

## Abstract

A daguerreotype image, the first commercialized photographic process, is composed of silver-mercury, and often silver-mercury-gold amalgam particles on the surface of a silver-coated copper plate. Specular and diffuse reflectance of light from these image particles produces the range of gray tones that typify these 19^th^ century images. By mapping the mercury distribution with rapid-scanning, synchrotron-based micro-X-ray fluorescence (μ-XRF) imaging, full portraits, which to the naked eye are obscured entirely by extensive corrosion, can be retrieved in a non-invasive, non-contact, and non-destructive manner. This work furthers the chemical understanding regarding the production of these images and suggests that mercury is retained in the image particles despite surface degradation. Most importantly, μ-XRF imaging provides curators with an image recovery method for degraded daguerreotypes, even if the artifact’s condition is beyond traditional conservation treatments.

## Introduction

Detailed, scientific analysis is becoming increasingly important in the preservation and interpretation of objects of cultural significance. The analysis of historical materials has traditionally required the removal of small, representative samples that are subsequently examined through a range of analytical techniques. However, sampling can alter or destroy the fragile specimens and may not provide results that warrant the destructive analysis^1^. The application of non-invasive techniques to the analysis of objects of cultural significance is becoming increasingly employed. X-ray methods in particular are making a large contribution to arts and archaeological research^2^. New applications are driven by a combination of advances in instrumentation applicable to synchrotron^3–5^ and non-synchrotron-based^6–10^ systems and synchrotron facilities are increasingly expanding to provide support for cultural heritage applications.

The presentation of daguerreotypes to the French Académie des Sciences in 1839 enabled photography to become a part of everyday life and captivated audiences with fine-detailed images that rivaled that of today’s digital cameras^11^. The process, invented by Louis-Jacques-Mandé Daguerre, started with a highly polished, silver-coated copper plate that was made light sensitive with iodine vapour. Once the scene of interest was exposed to the plate, the image was developed with heated mercury vapour, producing amalgamated silver-mercury image particles on the surface. Residual silver halides were removed with a sodium thiosulfate wash. An ancillary step was added two years later by Fizeau that involved pouring a gold-chloride-sodium thiosulfate solution over the plate, which was heated from below. This deposited gold on the image surface via an electroless deposition process^12–16^.

The daguerreotype image is created through specular and diffuse reflection of light from the silver-mercury-gold image particles on the surface. These image particles are formed when the photosensitized plate, which is covered with a silver halide (AgX), is exposed to reflected light from the object (e.g., an individual for a portrait). The interaction of light with the surface catalyzes the formation of silver particles^17^, on the plate surface in densities proportional to the intensity of light (eq. 1)^18^.

1 $$10{\rm{AgX}}+hv\to 2{{\rm{Ag}}}_{(5)}+5{{\rm{X}}}_{2}$$

**Figure 1:**
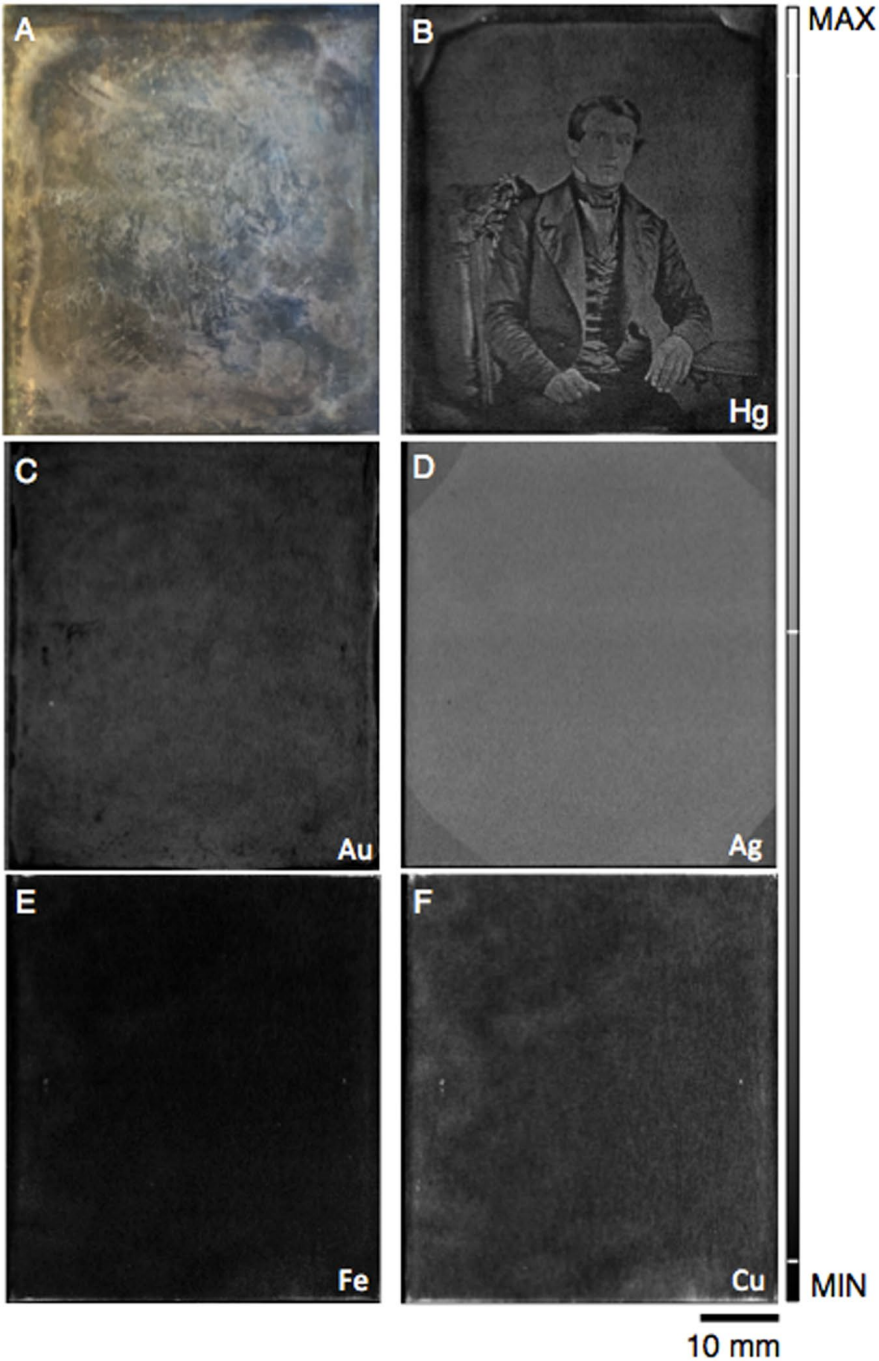
(**A)** An optical image of plate (PSC70:111) from a 19^th^ century daguerreotype from the National Gallery of Canada. (**B**–**F**) μ-XRF images of the Hg (Lα), Au (Lα), Ag (Lα), Fe (Kα), and Cu (Kα) distributions collected at Cornell High Energy Synchrotron Source (CHESS). Micro-XRF map for Hg collected at an incident energy of 13025 eV, a pixel size of 20 × 20 μm, and a working distance of 2 mm.

Intense light exposure produces high density (defined as the number of image particles per unit area) particle regions (~2 × 10^5^ particles/mm^2^) whereas little to no exposure to light yields regions moistly void of image particles (<100 particles/mm^2^)^19^. Furthermore, the image particles in high density and midtone regions have been reported to have diameters of 0.1–1 μm and 0.25–2.5 μm, respectively. An example of a high density image particle region would be the white collar of the man’s shirt (Fig. 1B). In contrast, shadow/dark tone parts (such as the man’s dark brown hair in Fig. 1B) contain particles ranging from 10–50 μm^19^. This spatial and size variations of image particles create the vast range of gray tones that are characteristic of daguerreotypes^20^.

Daguerreotypes are prone to tarnish. Fogging, white and blue-tinted haze, and black spots form as a result of reactions leading to the formation of silver compounds including oxides, chlorides, and sulfides^20^. Deterioration leads to significant reduction of image quality as it interferes with the Raleigh light scattering that produces the appearance of the fine details present in daguerreotype images. In extreme cases, the entire image appears to be lost such as in one of the daguerreotypes included here (Fig. 1A). Surface analysis has primarily identified the presence of silver sulfide, oxides, and chlorides to be the key contributors to image degradation^11,21^. The causes for daguerreotype image degradation could include a variety of factors including pre- and post-production conditions. Residual silver halides may reside on the plate from either the sensitizing step (eq. 2), or from the gilding step (as a gold-chloride-sodium thiosulfate was used), and/or from environmental contamination^18^.

2 $$2{{\rm{Ag}}}_{({\rm{s}})}+{{\rm{Cl}}}_{2({\rm{g}})}\to 2{\rm{AgCl}}$$

The presence of sulfur on the surface may arise for similar reasons, either during production, such as the gilding step, or during the “life” of the object by exposure to the environment. Typical indoor concentrations, such as in a museum, of sulfur and chlorine have been reported as H_2_S (0.3 ppb), COS (0.6 ppb), SO_2_ (30 ppb), HCl (0.4 ppb), and Cl_2_ (not detected)^22–25^.

**Figure 2:**
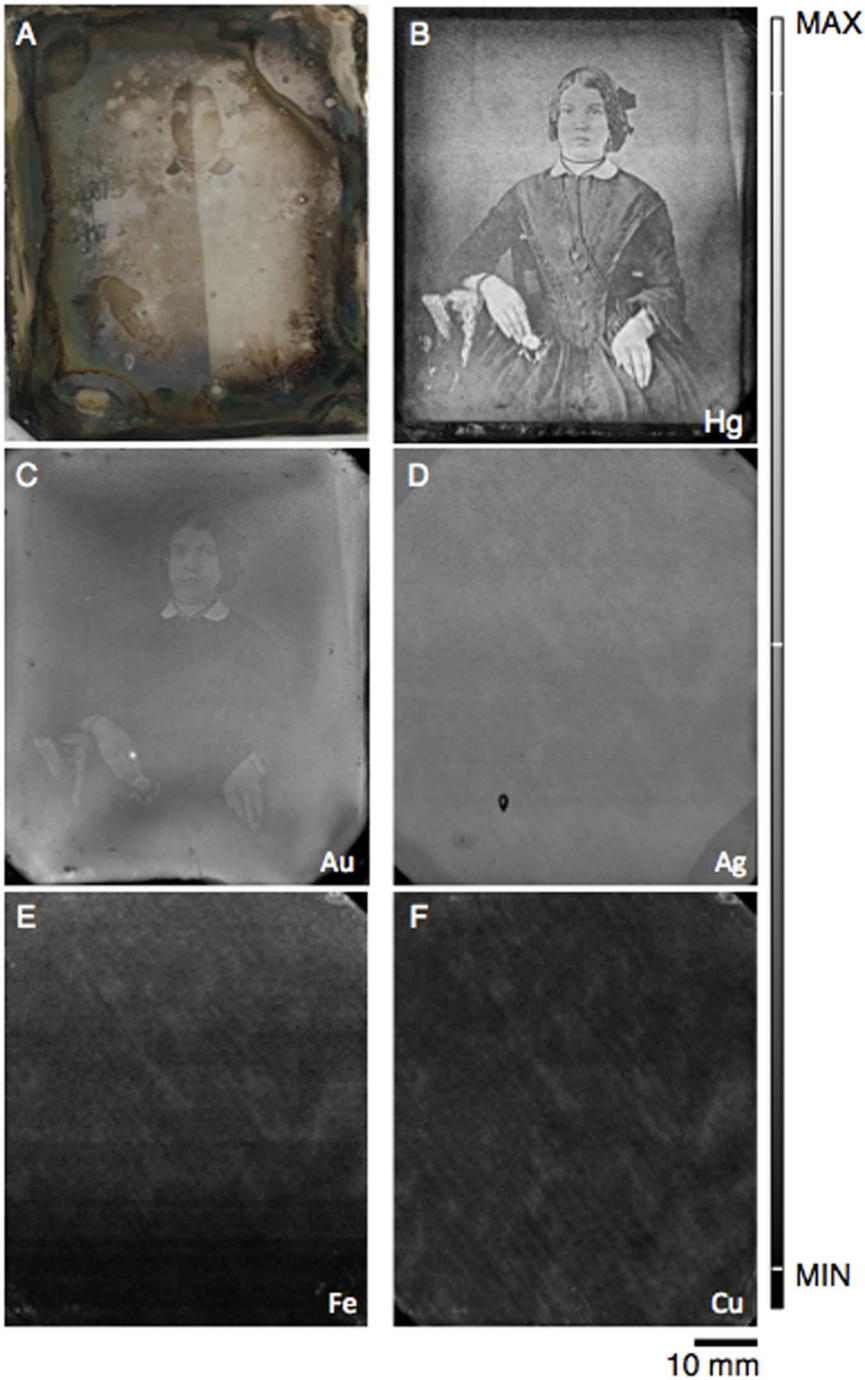
(**A)** An optical image of plate from a 19^th^ century daguerreotype from the National Gallery of Canada Study Collection. Optical image illustrates the effects of the use of chemical cleaning. The left half of the plate has been cleaned with a 1% NH_4_OH for 30 minutes. A small circular area produced by electrocleaning is visible on the optical image in the top left hand corner of the plate. These are preliminary and inconclusive conservation efforts; further investigation is required. (**B**–**F**) μ-XRF images of Hg (Lα), Au (Lα), Ag (Lα), Fe (Kα), and Cu (Kα) distributions collected at Cornell High Energy Synchrotron Source (CHESS). Micro-XRF maps for Hg collected at an incident energy of 13025 eV, a pixel size of 20 × 20 μm, and a working distance of 2 mm.

While portable X-ray Fluorescence (XRF) imaging setups have been successfully utilized in the analysis of cultural heritage objects, the spot size of all such instruments is too large for the investigation and imaging of daguerreotypes as the micron-level tonal variation dictates the requirement of a micron-sized beam for any XRF imaging (µ-XRF). Therefore, the research undertaken here used synchrotron radiation analysis, particularly μ-XRF imaging capabilities, to map the elemental distribution on the daguerreotype surface. Two original 19^th^ century plates from the National Gallery of Canada (NGC) were examined. One daguerreotype (Fig. 2A) has received preliminary conservation treatment while the second daguerreotype (Fig. 1A) has received no treatment and is completely unreadable to the naked eye. Using the Maia 384-element detector, which is optimized for high-speed operation and hence large area, high resolution elemental mapping^26,27^, the mercury (Hg) Lα fluorescence signal was imaged. Mercury was chosen as it plays an integral role in the production process and its distribution mirrors that of the image particles that make up the photograph. Through this rapid-scanning μ-XRF imaging capability, we demonstrate that mapping the Hg Lα fluorescence signal can retrieve entire lost images, despite severe degradation.

## Results

### Optical Examination

Optical imaging of the NGC daguerreotypes reveals the range of degradation of two historic plates (Figs 1A and 2A). It is apparent, due to surface tarnish that the images are beyond recognition (Fig. 1A) or are barely recognizable (Fig. 1B) when viewed by eye. Figure 1A shows weak outlines of the original portrait with the details of the face and setting indiscernible. The daguerreotype in Fig. 2A has tarnish along the perimeter of the image that contains dark degradation areas with oil-like residue. White fogging obstructs all portrait details. A difference in luster is observed between the right and left side of the image (Fig. 2A) as the left side of the plate was treated with a 30 minute, 1% NH_4_OH chemical dip to remove residual halides.

### Micro-XRF Imaging

The images of entire daguerreotype plates in the current study were obtained with a rapid-scanning Maia detector (described previously)^28^, which is capable of high count rates, exceeding 10^7^ photons/second^27^. The Maia data analysis software does not utilize regions of interest (ROIs) but instead fits a curve to each “electronic window” and to the background signal resultant from scatter. This enables both detection and discrimination of the Hg and gold (Au) L fluorescence signals over large scan areas in reasonable times in spite of the very large Cu excitation background signal and the lower energy resolution, which can be seen in Figure S2. The Hg/Au ratio for these samples averaged over the entire scan area is close to one. This means that any errors in the peak fitting resulting in elemental image cross contamination would be equally evident in both the Hg and Au XRF images. If there was cross contamination, a signal spike in the Hg image of Fig. 2A on the woman’s ring finger where the Au signal is markedly highest would be observed. As no such signal augmentation is present, the fitting routine is performing properly and no appreciable signal cross contamination is observed. While high count rates are not unique to the Maia detector, the images presented within this study greatly benefited from this feature, along with the Maia’s low dwell times and the micro-focused beam size.

Micro-XRF Hg distribution images from the two plates (Figs 1B and 2B) are shown alongside their optical images. The relative distribution of the other collected elements, which includes Fe, Cu, Au, and Ag, can be seen alongside the Hg fluorescence image in both Figs 1 and 2. While several plates from the NGC were analyzed for this study, only two plates are presented for clarity and are representative of the findings observed for all examined daguerreotypes.

Relative concentrations have been averaged from five dark regions and five light regions. Copper, along with Ag, shows the relatively greatest concentration of all collected elements. Iron, Ag, and Cu all show a decrease in relative concentration in the bright regions, which is due to the slight masking of Au and Hg. Conversely, Au and Hg have an increase in relative concentration in regions where image particle density is relatively greater. However, the Fe, Ag, Cu, and often Au µ-XRF images, do not provide any information regarding the nature of the image but solely for the substrate itself. It is only in the Hg image that the original image is observed. This is the result of the interaction between the heated Hg vapour and the charged Ag surface during daguerreotype processing that causes the distribution of Hg to follow that of the light exposure, and image particles, on the surface^13,15,29^. Therefore, the distribution of Hg on the surface represents the original image particle distribution. Although the daguerreotype optical image has been lost, the collection of the Hg L fluorescence allows the complete original image to be reconstructed.

## Discussion

The ability to view the Hg μ-XRF map despite the decline in optical integrity due to tarnish, suggests that relative Hg concentrations and distribution are preserved despite daguerreotype deterioration. This association of Hg with the original image is attributed to its critical role during daguerreotype production. The durability of Hg on the surface as well as the mechanism through which it amalgamates with the Ag particles is under debate in the literature^30,31^. To the best of the authors’ knowledge, there have been no proposed tarnish products involving Hg. However, recent work conducted by our group with synchrotron X-ray absorption near edge structure (XANES) spectroscopy has revealed the presence of mercury chloride and sulfide on the surface^32^. A possible dissociation of Hg with the original image may be caused from the de-alloying of the Ag-Hg amalgam. However, this process would not result in the mobilization of Hg from its original position and the relation between Hg and the image particles would still be observed by the fluorescence signal. Therefore, the specific chemistry of Hg does not matter for the image to be retained through its fluorescence signal, as this de-alloying process would not impact its relative position on the surface. However, if de-alloying has occurred and a plate undergoes a conservation treatment, the Hg may be dislodged and removed in solution, thereby unknowingly impairing the integrity of both the optical image and its potential recovery via XRF.

One such conservation treatment that may dislodge Hg from its intended location is electrocleaning. While the preliminary electrocleaning tests presented in this study (Fig. 1A) have shown no appreciable change to the Hg, Ag, and Au concentrations, these results do not provide substantive evidence to ascertain electrocleaning to be a safe treatment for daguerreotype conservation. Currently, an extensive electrocleaning study is underway by this team. Pre- and post-cleaning µ-XRF examination will be used to quantify the impact of this conservation method on the surface of the plate. Until that time, we suggest that electrochemical treatments be conducted with care.

Chemical dipping may also impact the relative concentrations of elements integral to the image. No division line is apparent in the Hg μ-XRF distribution image (Fig. 2B) from this chemical dip procedure, indicating that the Hg is not involved in the substitution reaction with the 1% NH_4_OH solution. However, elemental comparison of Hg and Au was conducted to compare the dipped and non-dipped side (Figure S2). When comparing like regions (non-dipped bright to dipped bright regions and non-dipped shadow to dipped shadow regions) across this conservation line no notable variation was apparent in Hg/Au ratios, suggesting that the complexing ability of the weak NH_4_OH solution only targets the residual halides, not the elements (Au and Hg) that are integral to the image. While the NH_4_OH dip does remove the white haze caused by residual silver halides, the daguerreotype image is far from recovered, highlighting the complexity of both the degradation and conservation techniques necessary for these 19^th^ century artifacts.

For this work, the incident beam was heavily attenuated to approximately 4 × 10^8^ photons/sec in the focal position to avoid saturating the detector due to the Cu substrate. This degree of attenuation suggests that mapping daguerreotypes may be well within reach with a high resolution, non-synchrotron source. An effort to develop a laboratory-based, portable XRF setup with a high count rate detector such as the Maia for imaging daguerreotypes is being considered for a long-term conservation effort. This would be beneficial for the *in situ* analysis of extremely fragile daguerreotypes not suitable for transportation to a synchrotron facility. However, we propose that using synchrotron µ-XRF mapping of the Hg L lines to image entirely tarnished daguerreotypes, especially those beyond conservation treatment, provides the best technique to preserve these important images of our history and is far superior to previously proposed methods. While a portable system may increase convenience, it will never match the scan size, step size, beamsize, dwell time, and the ability to select for an energy just above the Hg L_3_ edge of a synchrotron. Due to the 20 μm step size, some perceived blurriness may be noted in the µ-XRF images. To achieve a clarity comparable to that achieved by the naked eye, the step size would have to approach <10 μm. The statistical comparison of image resolution between the optical and fluorescence images is beyond the scope of this paper. However, even fine features such as the folds of the man’s tie and in the detailed carving of the high back chair in Fig. 1B as well as in the beaded bodice of the seated woman in Fig. 2B are visible on the Hg fluorescence images.

Davis and coauthors have previously used micro-focused XRF imaging to collect Au and Hg M line fluorescence from 19^th^ century daguerreotypes, producing a chemical reconstruction of the plates using a Rh source run at 50 kV^21^. A major challenge of µ-XRF imaging daguerreotypes is the excitation of Cu fluorescence from the substrate, as it produces an overwhelming background signal, masking the tiny fraction of Hg photons that are measured. The energy separation between the Au and Hg M lines (Au = 2122.9 eV; Hg = 2195.3 eV) is much smaller than that of the L lines (Au L_α1_ = 9713.3 eV; Hg L_α1_ = 9988.8 eV), indicating that these signals can be more easily distinguished and fitted using L emission^33^. Davis and colleagues were able to produce a replicate image from their post-processing technique. However, they were examining daguerreotypes that had hardly any tarnish and nearly every sign of physical damage such as scratches are clearly visible in their XRF images. If their methodology were used in this current study, their mode of imaging would not be possible due to the severity of the corrosion on the daguerreotypes analyzed in this study (Fig. 1A). For instance, factoring in all the differences in scan size, step size, dwell time etc., the scans presented in this work is over 60000 times faster than that proposed by Davis. The ability to produce a µ-XRF scan with excellent clarity in a timely manner is a fundamental advancement in this area of research. To reiterate this point, representative figures acquired from a bright region (white collared shirt) and a dark region (dark vest) from daguerreotype PSC70:111 are shown in Fig. 4. Here, the energy window has been restricted to the range 7.5–11 keV to allow for the deconvolution of peaks associated with Au Lα and Hg Lα within these contrasting areas. Comparing the XRF spectra from the dark vest (spectrum A) and the white collared shirt (spectrum B), a clear difference between the Au Lα and Hg Lα is observed. The bright area (spectrum B) shows a significant increase in Hg signal, due to the increase in density of image particles in highlight regions. No substantial variation between the Au fluorescence in the two spectra is expected due to the absence of a Au image (Fig. 1D).

Recent work by Kozachuk *et al*.^32^ used synchrotron-based XANES spectroscopy and µ-XRF microscopy to examine a collection of tarnished 19^th^ century daguerreotypes to characterize the tarnish products on the image surface. Not only did this work reveal information regarding the distribution of tarnish on the plate surface, but it demonstrated that synchrotron radiation analysis did not induce any noticeable negative optical or chemical effects due to absorbed dose, a crucial consideration when undertaking conservation science analysis. The absorbed dose determined by the experimental parameters used by Kozachuk *et al*.^32^ (average X-ray energy = 3350 eV; incident flux = 2 × 10^9^ photons/sec; exposure time = 90 min/region; region = 25 μm × 25 μm pixel) was over 28 million times higher, indicating that the parameters used in the current study will not impose any negative alterations to the daguerreotypes.

A one-dimensional scan of the Hg L fluorescence across the chest of the seated man (Fig. 1a) is shown in Fig. 5. The scan, which tracks the Hg signal from the man’s right lapel (position 0 μm), vest (position 5000 μm), white shirt (7500 μm), vest (11300 μm), and left lapel (12000 μm), shows how Hg varies across the different grey tones. The greatest signal is observed across the white shirt with peaks in intensity at the edges of the jacket where highlights are observed in the image. This confirms the correlation between Hg and the image particles that increase in density where white areas are detected. Examination of the counts in adjacent positions within this line scan shows a significant variation in the Hg counts at contrast boundaries. For example, the Hg counts at the highlight of the man’s right lapel is 91514.4 while the adjacent dark pixel of the vest has counts of 7618.9. In contrast, within the vest at neighbouring pixels, the Hg counts were 3443.1 and 3063.5. The ratio of maximum to minimum signal along the selected line of pixels is reported as 4130.

Similar to these previous findings^32^, no beam damage resulting from synchrotron analysis was observed in this study, either visibly or through post-synchrotron scanning electron microscopic analysis. The absorbed dose for each sample during the XRF scan was determined to be ~40 Gy with the air between the daguerreotype and the detector acting as a conductive mechanism for heat removal, if any is present. While soft matter samples (such as textiles, dentine, and bone) are at a higher risk for beam damage, hard materials, such as silver, are less prone to radiation-induced damage due to their thermal and electrical conductivity. This does not suggest that damage to hard matter samples with modern synchrotron sources is impossible^34,35^, but that if damage is to occur, it is typically found with prolonged (i.e., scale of hours versus 4 msec used in this study) exposure to a beam in the same spot. Another factor that can possibly induce beam damage to hard materials is the use of extremely brilliant beams (i.e. >4 orders of magnitude more brilliant than the beam used at the G3 line) paired with exposure times far longer than the two daguerreotypes experienced during µ-XRF collection. Moreover, reports of beam damage to hard material compounds often focus on semiconductor substrates like silicon nitride and borosilicate glasses and not on highly conducting metal such as silver^35^. Damage to heritage objects is much more likely in studies looking at paintings under paintings^5^ where the paint is far less conductive than silver metal. However, none of those studies suggest that those exceedingly valuable paintings were damaged in any way. Thus, these combined factors would suggest that the chance of sample damage to daguerreotypes is exceedingly small. In sum, we can conclude with a fair degree of certainty that no damage was induced on the 19^th^ century plates from this work despite its success in recovering images masked by tarnish.

## Conclusions

The innovative contribution of this research is the use of μ-XRF mapping using the Hg L emission lines and the Maia 384-element detector to analyze an entire daguerreotype plate in a non-destructive, non-invasive, and non-contact manner. The Hg map reveals fine features within the original images, even when degradation and tarnish concealed the original image from optical view. The ability to recover lost images will enable museums to expand their understanding of daguerreotype collections, as severely degraded plates would not otherwise have been able to be studied or viewed by interested scholars. Furthermore, an established method of cleaning by means of a weak NH_4_OH solution, appeared to have no negative impact on the concentration and distribution of Hg. However, as no pre- and post-dip analysis was conducted, no definitive conclusions regarding the efficacy of this treatment can be made; this is currently under examination. Further investigation of the established electrocleaning methodology needs to be undertaken to ensure future conservation treatment does not negatively impact the daguerreian surface and the image particles.

## Methods

### Sample Selection and Preparation

The two daguerreotypes analyzed in this study were provided by co-author John McElhone from the Canadian Photography Institute at the National Gallery of Canada (NGC). These two plates were chosen as they display a range of tarnish features typical of historic daguerreotypes and were available for loan. The NGC acquired these two daguerreotypes through casual purchases and donations with the intent that they be used for study purposes. John McElhone removed the plates from their original cases prior to being received for analysis. Daguerreotype Fig. 2A is from the NGC Research Study Collection and Fig. 1A is from the NGC collection (accession number PSC70:111). Both of these images have substantial tarnish on the surface, observed as a white haze that covers the image. No sample preparation was conducted prior to analysis other than the preliminary electrocleaning and chemical dipping attempts.

### Synchrotron micro-XRF Spectroscopy

The synchrotron experiments were conducted on the G3 line at the Cornell High Energy Synchrotron Source (CHESS), Cornell University, Ithaca, NY, U.S.A. The incident energy was set to 13025 eV, above the Hg L_3_ edge at 12284 eV, in order to excite fluorescence from the Hg L emission lines. At this incident energy of 13025 eV, the energy resolution was ~1.4 eV. The incident beam was heavily attenuated to approximately 4 × 10^8^ photons/sec in the focal position to avoid saturating the detector. Incidentally this will also reduce the chance of radiation damage. The working distance was 2 mm between the detector and the daguerreotype. Micro-XRF spectroscopy elemental maps were collected using the Maia-384 element detector by continuously scanning the sample (called fly-scan mode), while the detector and the beam remain fixed^36,37^. The low-energy cut-off of the detector is limited to approximately 2 keV and the energy resolution of the detector is 280 eV at the Mn K_α_ line, which allows the separation of the Hg Lα from that of Au.

The daguerreotype was posteriorly secured within a metal frame on two-sided Kapton tape while the corners of the photograph were cradled with non-adhesive Kapton film. A 70 × 80 mm scan range was analyzed with an estimated dwell time of 4 ms per 20 × 20 μm pixel over a collection time of 8.5 h. The beamsize was 20 × 30 μm. The flat surface of the daguerreotype afforded optimal conditions for data collection and μ-XRF modeling with respect to the calculation of X-ray fluorescence yields. Elemental concentrations are considered to be semi-quantitative. The elemental maps are shown with the observed counts and the maximum intensity threshold adjusted to best represent the high dynamic range data in a single image. The elements of interest were Ag, Au, Hg, Fe, and Cu.

### Data processing of synchrotron micro-XRF images

**Figure 3:**
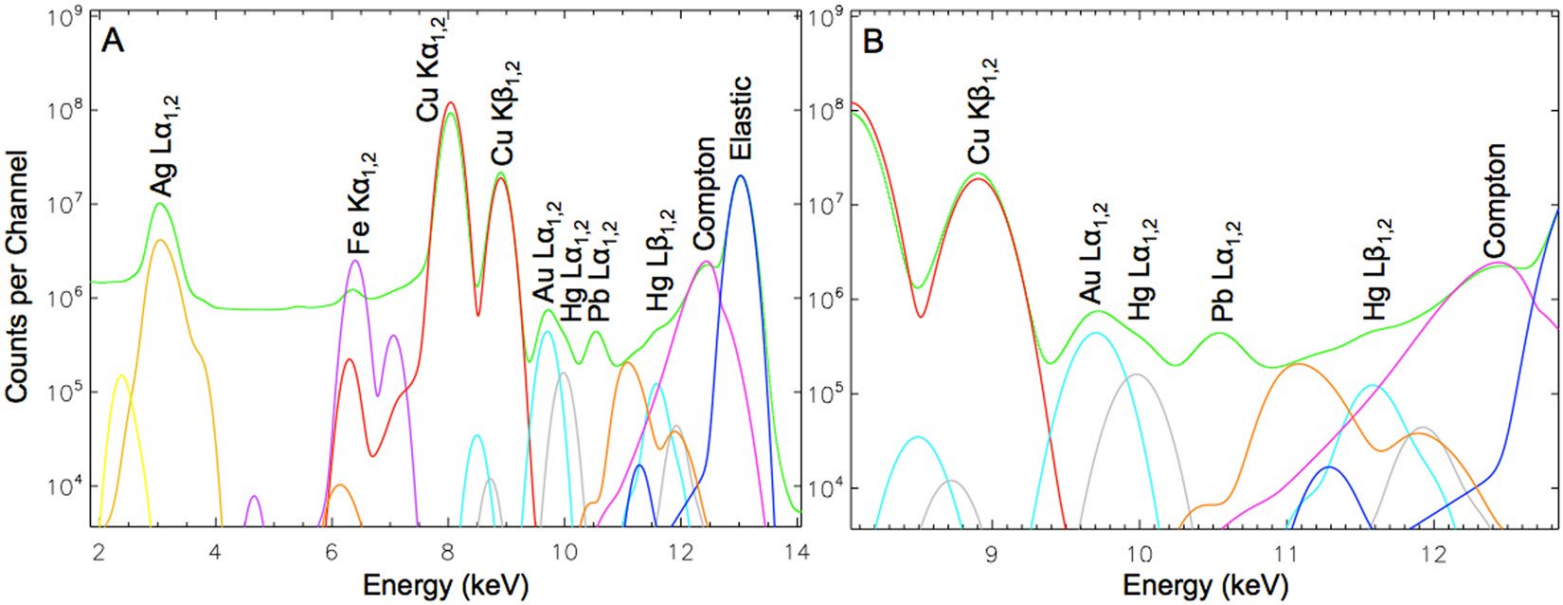
(**A)** An example of spectral decomposition from the XRF spectrum of a 19^th^ century daguerreotype from the National Gallery of Canada (PSC70:111). The green curve represents the Maia data with the individual fits represented by other colors. (**B)** An enlarged region that focuses on the energy range 8–13 keV. Both spectra include the identification of key elements. The green curve represents the Maia data with the individual fits represented by other colors.

The synchrotron μ-XRF data was processed using GeoPIXE. Elemental concentration maps were produced using the fundamental parameters dynamic analysis (DA) approach^38^. The sample matrix was approximately modeled with reference to Au foil (density = 19.32 g/cm^3^). Any deviations between the examined daguerreotypes and this Au reference do not significantly affect the ratios of the elemental concentrations examined within this work. All spectra include lead (Pb) and molybdenum (Mo) fluorescence (which are weak since the excitation energy is below the Mo K edge and the Pb L edges), which are from the beamstop and from inside the Maia detector, respectively^27^. An example of a summed spectrum with the associated fit can be seen in the Fig. 6 with a deconvoluted spectrum presented in Fig. 3. Elements of interest are selected for the fit, which then appear as individual peaks (multi-colored lines) under the Maia data (green line) in the deconvoluted spectrum (Fig. 3). To acquire the spectrum presented in Fig. 3, an energy calibration in GeoPIXE is completed. This ensures that all 384 pixels of the Maia detector are reporting each fluorescence peak at the same energies. As mentioned, a Au calibration foil was used to generate the calibration standard for the beam energy used in this experiment. The Au calibration applies the new coefficients required to correct for any offsets in the energy scale (x-axis) to each pixel spectrum. This newly calibrated data can now be summed into a single spectrum. The subsequent fitting process involves reiterating all and/or some of the following steps: (1) the selection of elements relevant to the sample and their emission lines (i.e., K, L, and M); (2) ensure that the signal filter is accounted for (in this case 5 mm of air); optimize the fit of the background and the elastic and Compton scattering peaks; restriction of the full width half max (FWHM) of the peak fitting and the adjustment of the low energy tails. Cycling through this process produces the ideal dynamic analysis (DA) matrix from which the elemental maps are generated. Relative trace element concentrations of Hg and Au were determined by selecting regions of interest (i.e., the collar on a dress for a highlight region versus the hair of an individual for a dark region) from the collected elemental maps. This generated a table of elemental concentration values that were then exported from GeoPIXE. Within each image, five dark areas and five bright areas were selected and the Hg/Au ratio was conducted to provide a comparison of Au and Hg levels across different regions of the plate. These values, along with the locations of the five light (L1-L5) and five dark (D1-D5) regions used for the ratio calculations, are shown in Figure S1 and Figure S2 (Supplementary Materials). A comparison between Hg/Au values from NH_4_OH dipped and non-dipped side is included (Figure S3).

**Figure 4:**
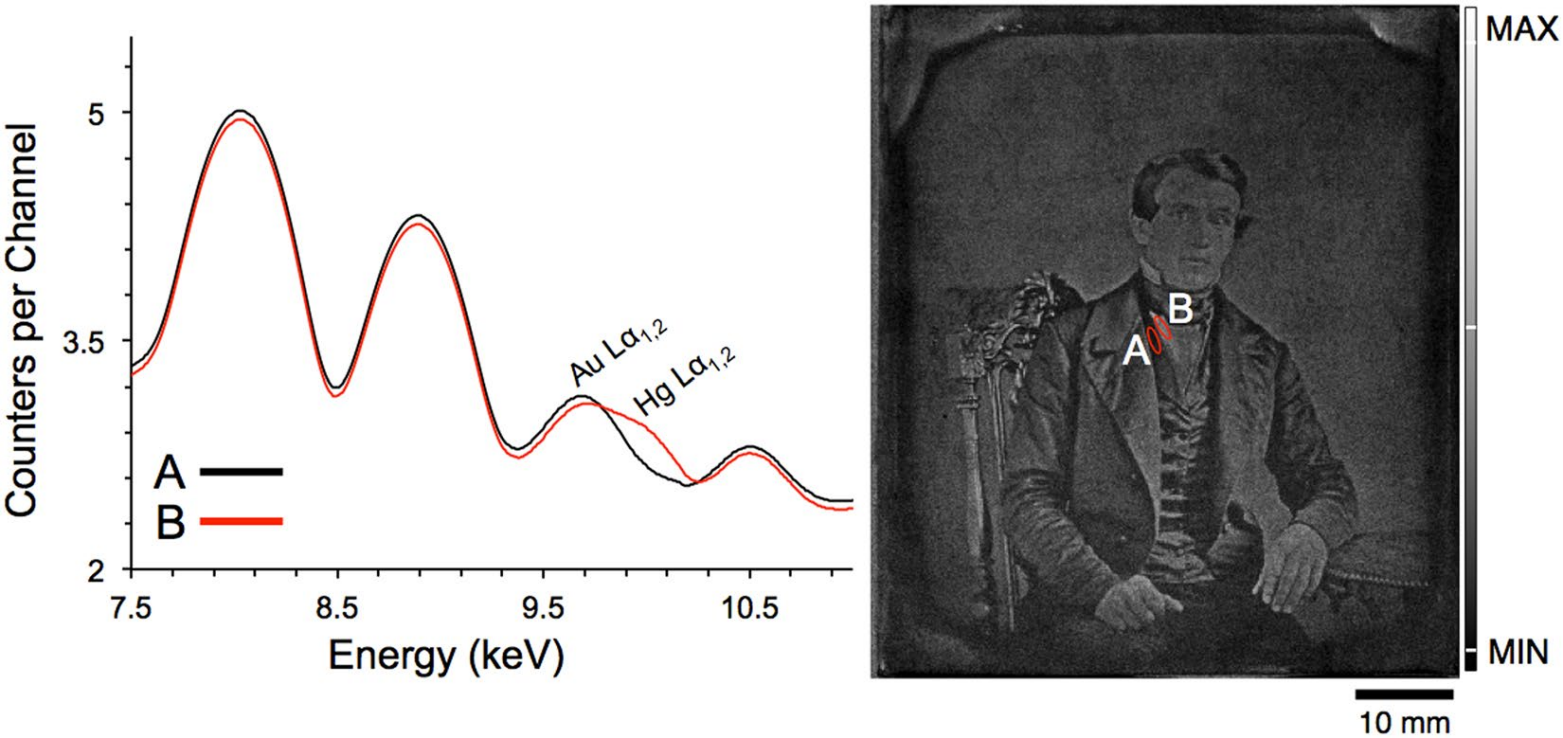
Spectral decomposition from the XRF spectrum collected at a bright region (white collared shirt) and a dark region (dark vest) of a 19^th^ century daguerreotype from the National Gallery of Canada (PSC70:111).

**Figure 5:**
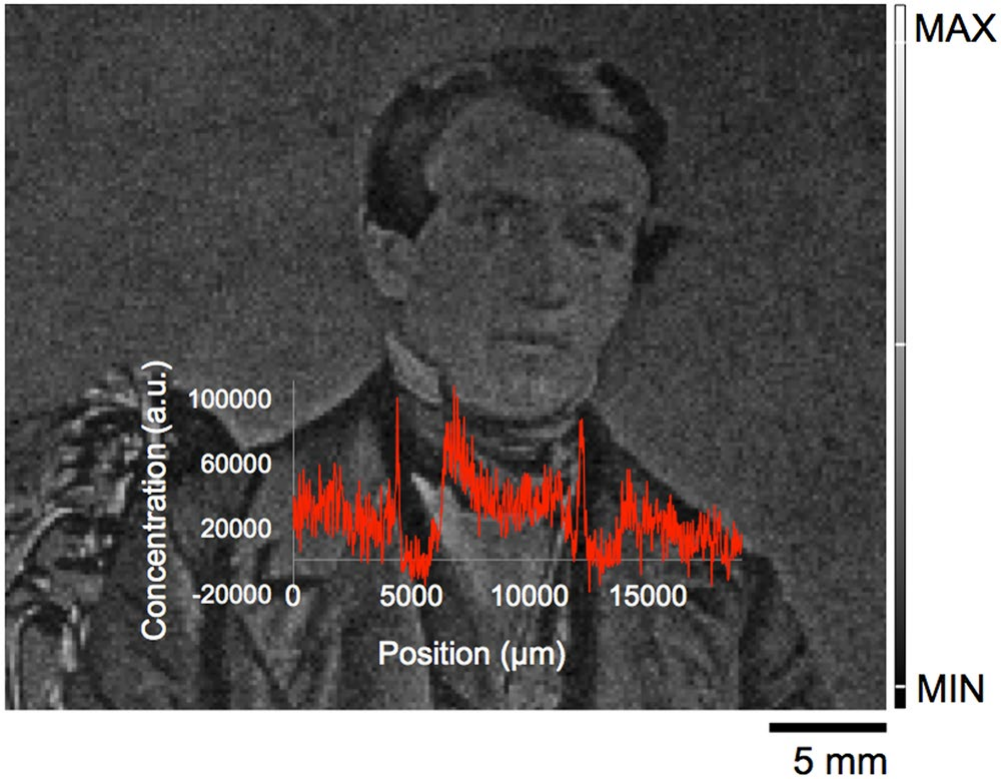
One-dimensional scan on the linear scale of Hg L fluorescence across the jacket lapel, vest, and white collared shirt of 19^th^ century daguerreotype (PSC70:111).

**Figure 6:**
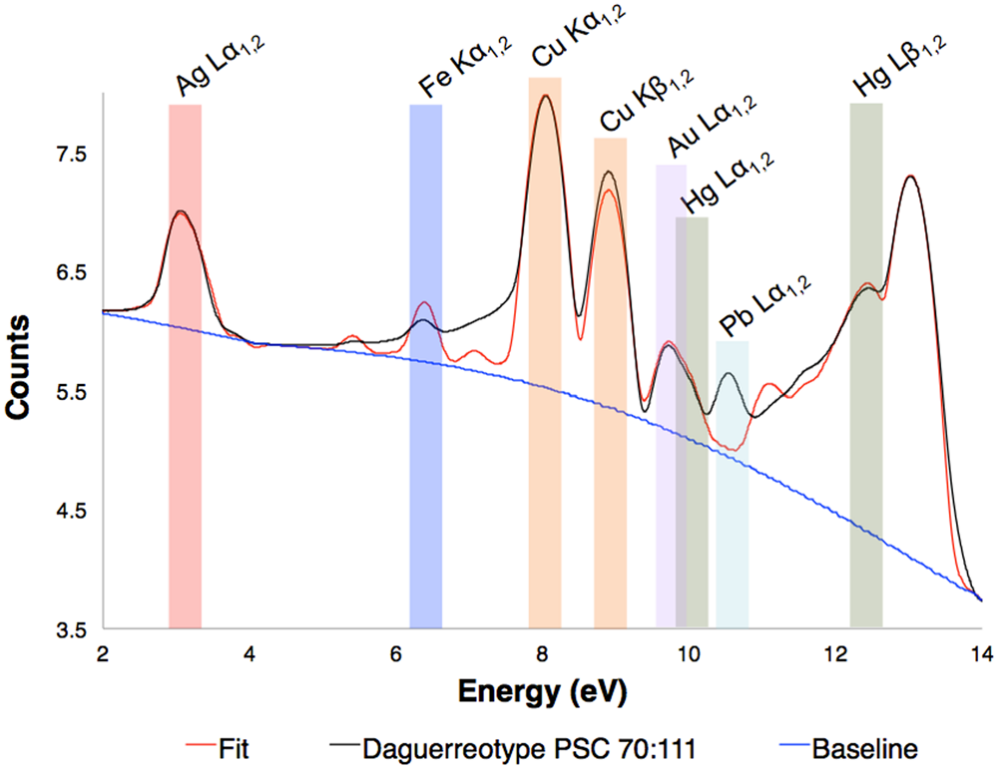
Micro-XRF spectrum for daguerreotype PSC70:111 (Fig. 1) that has undergone spectra summation, calibration, and fitting in GeoPIXE. Elements of interest are highlighted. Energy resolution of the detector is 280 eV.

### Preliminary conservation testing

The chemical dip for plate Fig. 2A was performed at The University of Western Ontario following standard procedures to clean these plates. A 1% NH_4_OH solution at room temperature (21 °C) was used for 30 minutes to remove residual halides (X = Cl^−^, Br^−^, I^−^) that cause plate clouding (eq. 3)^39^.

3 $${\rm{AgX}}+2{{\rm{NH}}}_{4}{\rm{OH}}\to {\rm{Ag}}{({{\rm{NH}}}_{3})}_{2}^{+}+{{\rm{X}}}^{-}+{{\rm{H}}}_{2}{\rm{O}}$$

Any residual halides would depend on the original production process. The plate was subsequently thoroughly rinsed with deionized water and dried in a stream of dry Ar gas.

Preliminary electrochemical treatments were completed using a 10 mm diameter cell filled with 0.1 M NaNO_3_ electrolyte at room temperature (21 °C)^40^. The potential was scanned from the open circuit value down to −1.5 V at a scan rate of 10 mV·s^−1^ over a scan range of 1 V. All potentials were measured and quoted against a silver-silver sulfate (Ag/Ag_2_SO_4_) saturated sulfate reference electrode that has a potential of 0.318 V with respect to the saturated calomel electrode. The open circuit potential was recorded for 600 s before and after each potentiodynamic sweep. The spot that was touched by the electrocleaning cell can be seen as a dark spot on the top right hand corner of the plate in Fig. 2A.

## Electronic supplementary material

Supplementary Material
