# Author Correction: Recovery of Degraded-Beyond-Recognition 19^th^ Century Daguerreotypes with Rapid High Dynamic Range Elemental X-ray Fluorescence Imaging of Mercury L Emission

**DOI:** 10.1038/s41598-018-30576-6

**Published:** 2018-08-09

**Authors:** Madalena S. Kozachuk, Tsun-Kong Sham, Ronald R. Martin, Andrew J. Nelson, Ian Coulthard, John P. McElhone

**Affiliations:** 1 The University of Western Ontario, The Department of Chemistry, 1151 Richmond Street, London, Ontario, N6A 5B7 Canada; 2 The University of Western Ontario, The Department of Anthropology, 1151 Richmond Street, London, Ontario, N6A 5C2 Canada; 3 Canadian Light Source Inc., 44 Innovation Boulevard, Saskatoon, SK S7N 2V3 Canada; 4 National Gallery of Canada, Musée des beaux-arts du Canada, 380 Sussex Drive, P.O. Box 427, Station A, Ottawa, Ontario, K1N 9N4 Canada

Correction to: *Scientific Reports* 10.1038/s41598-018-27714-5, published online 22 June 2018

This Article contains typographical errors in the Methods Section.

“At this incident energy of 13025 eV, the energy resolution was ~1.4 eV.”

should read:

“At this incident energy of 13025 eV, the experimental setup made use of a multilayer monochromator with an energy resolution of ~80 eV.”

“The sample matrix was approximately modeled with reference to Au foil (density = 19.32 g/cm^3^).”

should read:

“The sensitivity of the measurement, allowing the determination of elemental concentration from measured X-ray fluorescence counts, was calibrated using an Au foil (thickness = 9.94 nm; density = 19.32 g/cm^3^).

