# Supplementary material for: Recovery of Degraded-Beyond-Recognition 19th Century Daguerreotypes with Rapid High Dynamic Range Elemental X-ray Fluorescence Imaging of Mercury L Emission
Source: Sci Rep. 2018 Jun 22;8:9565. doi: 10.1038/s41598-018-27714-5 (PMC6015064; doi:10.1038/s41598-018-27714-5)
Supplement: Supplementary file 1 — Supplementary Material [file 41598_2018_27714_MOESM1_ESM.doc]

**Supplementary Material**

**Recovery of Degraded-Beyond-Recognition 19^th^ Century Daguerreotypes with Rapid High Dynamic Range Elemental X-ray Fluorescence Imaging of Mercury L Emission**

**Madalena S. Kozachuk^1^, Tsun-Kong Sham^1^*, Ronald R. Martin^1^, Andrew J. Nelson^1,2^, Ian Coulthard^3*^, John P. McElhone^4^**

^1^The University of Western Ontario, The Department of Chemistry, 1151 Richmond Street, London, Ontario, N6A 5B7, Canada

^2^The University of Western Ontario, The Department of Anthropology, 1151 Richmond Street, London, Ontario, N6A 5C2, Canada

^3^Canadian Light Source Inc., 44 Innovation Boulevard, Saskatoon, Saskatchewan, S79, 2V3

^4^National Gallery of Canada, Musée des beaux-arts du Canada, 380 Sussex Drive, P.O. Box 427, Station A, Ottawa, Ontario, K1N 9N4, Canada

*Correspondence to: tsham@uwo.ca (T.K. Sham); Ian.coulthard@lightsource.ca

**Figures**


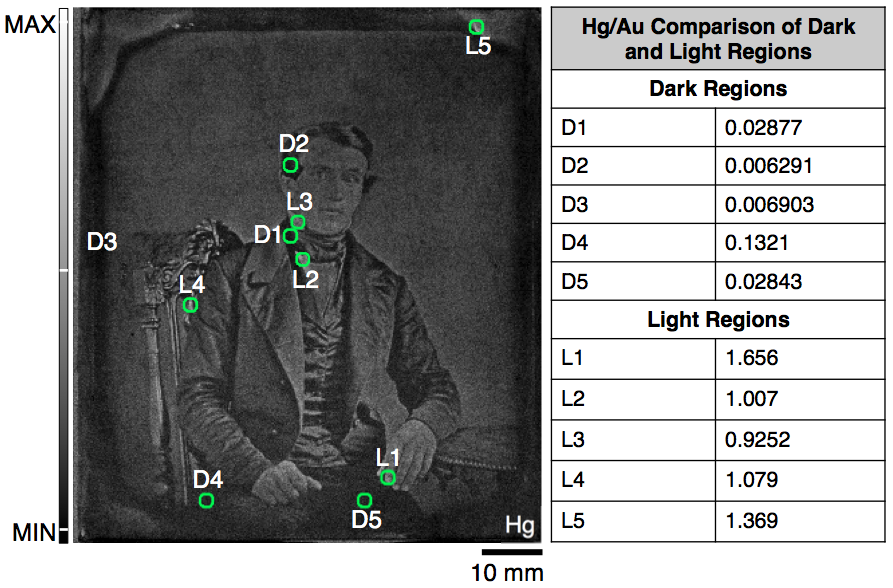


**Figure S1.** Micro-X-ray fluorescence (μ-XRF) image of Hg (Lα_1,2_) collected at Cornell High Energy Synchrotron Source (CHESS) of plate (PSC70:111) from the National Gallery of Canada. Locations where elemental concentrations were examined are marked by green circle. Relative average concentrations were calculated from the counts collected at each of the chosen sites. Five locations were chosen from dark areas (D1 – D5) and from bright regions (L1 – L5). The ratios from Hg (Lα_1,2_)/Au (Lα_1,2_) are presented in the table to the right of the μ-XRF image.


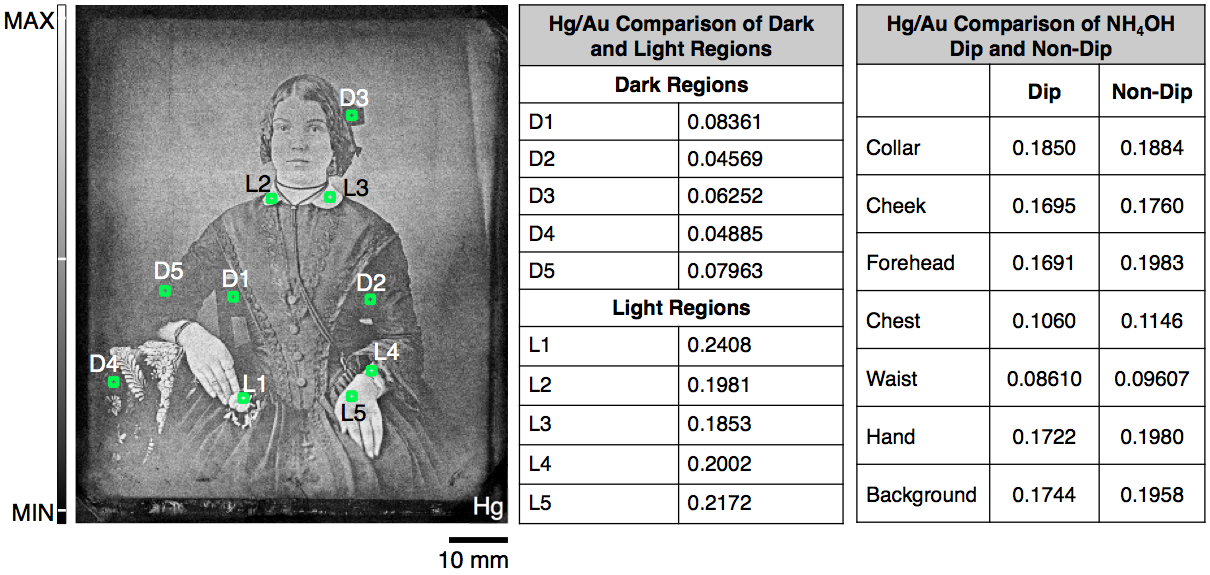


**Figure S2.** Micro-X-ray fluorescence (μ-XRF) image of Hg (Lα_1,2_) of the Study Collection plate from the National Gallery of Canada collected at Cornell High Energy Synchrotron Source (CHESS). Locations where elemental concentrations were examined are marked by green circle. Relative average concentrations were calculated from the counts collected at each of the chosen sites. Five locations were chosen from dark areas (D1 – D5) and from bright regions (L1 – L5). The ratios from Hg (Lα_1,2_)/Au (Lα_1,2_) are presented in the table to the right of the μ-XRF image. A comparison between Hg/Au values from the chemical dipped and non-dipped sides is included. Not all examined regions are marked. Due to a fixed lighting source, variation in Hg values is most likely due to slight differences in incident light intensity. Subsequently, ratio values will be impacted. Although the chemical dipped half shows a variation from the values collected from the non-dipped half, further analysis should be conducted examining the same areas before and after NH_4_OH dipping.


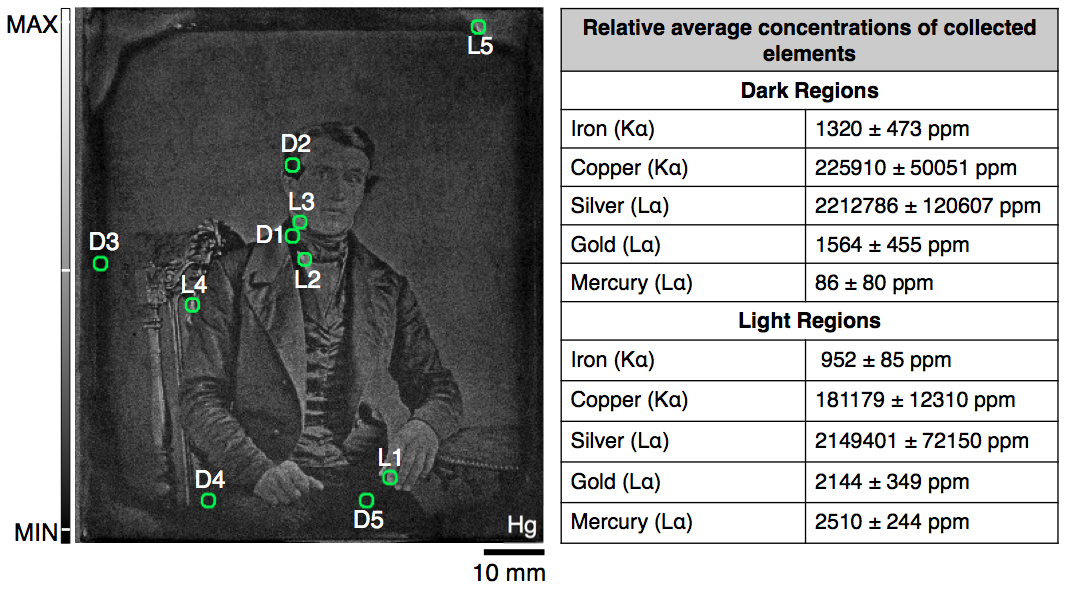


**Figure S3.** Micro-X-ray fluorescence (μ-XRF) image of Hg (Lα_1,2_) collected at Cornell High Energy Synchrotron Source (CHESS) of plate (PSC70:111) from the National Gallery of Canada. Locations where elemental concentrations were examined are marked by green circle. Relative average concentrations were calculated from the counts collected at each of the chosen sites for iron (Kα), copper (Kα), silver (Lα), gold (Lα), and mercury (Lα). These values are presented in the table to the right of the μ-XRF image along side their standard error. Five locations were chosen to average from dark areas (D1 – D5) and from bright regions (L1 – L5).
